# Cardio- and neuroprotective effects by pretreatment of dietary moringin from *Moringa oleifera* seeds and α-CD/moringin formulation in a rat model of isoproterenol-induced myocardial infarction

**DOI:** 10.1017/jns.2025.10035

**Published:** 2025-09-04

**Authors:** Ahmad Faizal Abdull Razis, Ramla Muhammad Kamal, Gina Rosalinda De Nicola, Sabine Montaut, Enoch Kumar Perimal, Hafandi Ahmad, Patrick Rollin, Sébastien Rigaud, Emanuela Mazzon, Florence Djedaini-Pilard

**Affiliations:** 1 Department of Food Science, Faculty of Food Science and Technology, Universiti Putra Malaysia, 43400 UPM Serdang, Selangor, Malaysia; 2 Natural Medicines and Products Research Laboratory, Institute of Bioscience (IBS), Universiti Putra Malaysia, 43400 UPM Serdang, Selangor, Malaysia; 3 Laboratory of Food Safety and Food Integrity, Institute of Tropical Agriculture and Food Security (ITAFoS), Universiti Putra Malaysia, 43400 UPM Serdang, Selangor, Malaysia; 4 Department of Pharmacology, Federal University Dutse, 720101 Dutse, Jigawa State, Nigeria; 5 Research Centre for Vegetable and Ornamental Crops, Council for Agricultural Research and Economics (CREA), Via dei Fiori 8, 51017 Pescia, Italy; 6 Biomolecular Sciences Programme, School of Natural Sciences, Laurentian University, 935 Ramsey Lake Road, P3E2C6 Sudbury, ON, Canada; 7 Curtin Medical School, Faculty of Health Sciences, Curtin University, Kent St, Bentley WA 6102, Australia; 8 Department of Biomedical Sciences, Faculty of Medicine and Health Sciences, Universiti Putra Malaysia, 43400 Serdang, Selangor, Malaysia; 9 Department of Veterinary Preclinical Sciences, Faculty of Veterinary Medicine, Universiti Putra Malaysia, 43400 Serdang, Selangor, Malaysia; 10 ICOA, CNRS, Université d’Orléans, UMR 7311, BP 6759, F-45067 Orléans, France; 11 LG2A UMR 7378, Université de Picardie Jules Verne, 33 rue Saint Leu - UFR des Sciences, F-80039 Amiens, France; 12 Department of Medical, Oral and Biotechnological Sciences, University “G. D’Annunzio” Chieti-Pescara, 66100 Chieti, Italy

**Keywords:** α-CD/moringin, Cardioprotection, Glucomoringin, Isoproterenol myocardial infarction, Moringin, Neuroprotection

## Abstract

The aim of this study was to investigate the cardio- and neuroprotective effects of moringin (MG), a dietary isothiocyanate readily derived from *Moringa oleifera* seed, in a rat model of isoproterenol (ISP) induced myocardial infarction (MI). Thirty-two adult male Sprague Dawley rats were divided into 4 groups: a control group, an MI group, a group pretreated with freshly prepared MG solution (MG + MI; glucomoringin 20 mg/kg + 30 µl myrosinase/rat), and a group pretreated with a stable α-cyclodextrin-based formulation of MG (α-CD/MG + MI, 42 mg/kg). Pretreatment was administered daily for 7 days. On days 6 and 7, rats received ISP (85 mg/kg, subcutaneously) at 24-hour interval. MI rats exhibited impaired hemodynamic and behavioural responses, marked elevation of malondialdehyde (MDA), and reduced activity of the antioxidant enzymes superoxide dismutase (SOD) and catalase (CAT) in both myocardial and hippocampus tissues. MI rats also demonstrated a significant rise in serum cardiac biomarkers, including cardiac troponin I (cTnI) and creatine kinase myocardial band (CK-MB). In contrast, pretreatment with MG and α-CD/MG significantly improved locomotor and exploration behaviour, reduced heart rate (HR), and enhanced mean arterial pressure (MAP). Furthermore, both treatments lowered serum cardiac markers, restored redox balance, normalised brain monoamines levels, and improved the histoarchitecture of myocardial and hippocampus tissues. These findings suggested that MG and α-CD/MG exert cardioprotective and neuroprotective effects by attenuating oxidative stress in a rat model of ISP-induced MI. Overall, intake of MG and α-CD/MG may represent a potentially effective pretreatment strategy for mitigating the systemic perturbations associated with myocardial infarction.

## Graphical abstract

**Figure:**
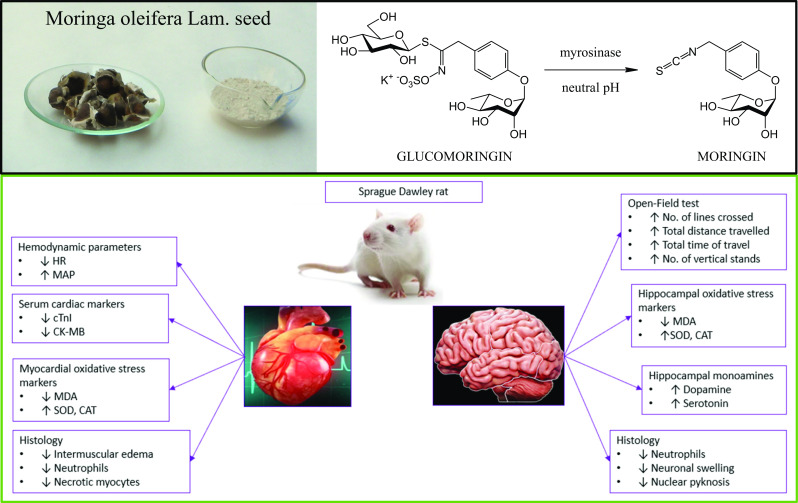

## Introduction

Myocardial infarction (MI) is the main cause of death and a major worldwide public health issue, nowadays.^(1)^ MI refers to the necrosis of cardiac myocytes resulting from complete occlusion of a coronary artery. Usually, MI is the outcome of the rupture of coronary atheromatous plaque with the affected myocardial area becoming necrosed from oxidative stress-induced injury, producing leakage of cardiac markers and decline of myocardial functioning. These cardiac markers include cardiac troponins (cTn) and creatine kinase myocardial band (CK-MB) that are used as MI diagnostic markers.^(2)^ Various hemodynamic, biochemical and morphological alterations occur and moreover, behavioural anomalies, like anxiety symptoms, manifest as a consequence of brain-heart interactions.^(3)^ This can be caused by increased generation of reactive oxygen species (ROS) in the frame of high basal oxygen consumption and low levels of antioxidant enzymes. Furthermore, the synthesis of monoamines like dopamine and serotonin is altered as a consequence of acute stress.^(4)^ Similar to the myocardium, the morphological architecture of the brain is affected and become distorted, as well.^(5)^ Currently, cardiovascular and behavioural symptoms of MI are treated independently, using multiple drugs.^(6)^ The World Health Organization has prompted for further research to generate more efficient treatment options. Several natural alternatives derived from medicinal plants have long proven effectiveness in many heart and neuronal diseases treatment.^(7)^

**Figure 1.**
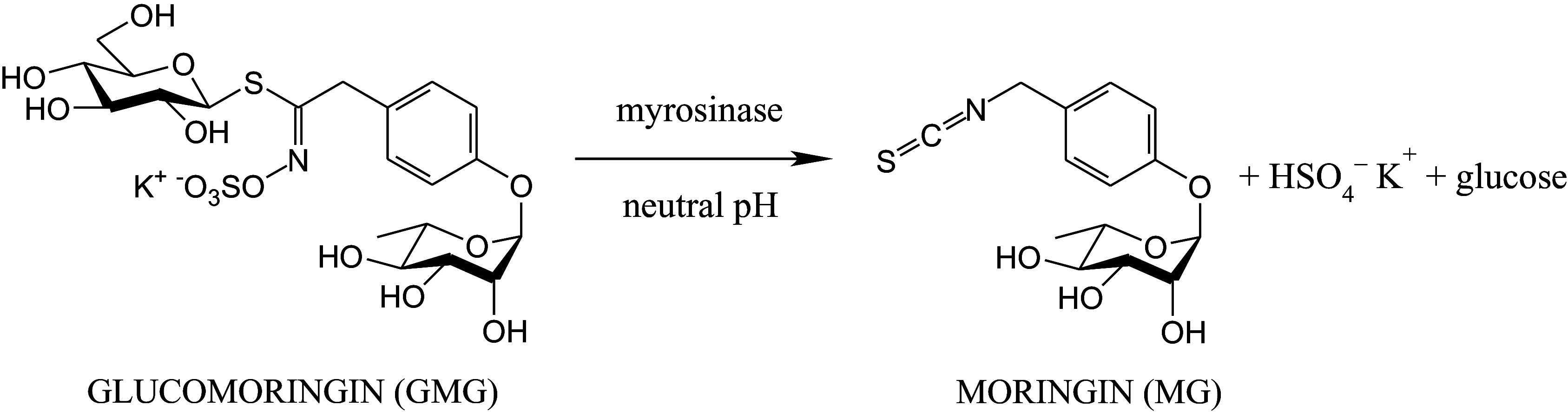
Production of 4-(*α*-L-rhamnosyloxy)benzyl isothiocyanate (moringin; MG). Reaction of myrosinase (*β*-thioglucoside glucohydrolase; EC 3.2.1.147) catalysed hydrolysis of 4-(*α*-L-rhamnosyloxy)benzyl glucosinolate (glucomoringin; GMG), purified from *Moringa oleifera* seeds, in buffered solution at neutral pH to produce MG.

*Moringa oleifera* Lam. (*Moringaceae*), often referred to as the miracle tree, contains high amount of bioactive nutrients and dietary antioxidants, which help in ameliorating oxidative stress and degenerating diseases. Native to northern India, *M. oleifera* is now widely distributed and cultivated across tropical and subtropical climates worldwide. Current and ongoing research has revealed *M. oleifera* as a significant tree with multifunctional applications. All parts of the plant, especially leaves and seeds, are used in human and animal nutrition added to food preparation as a supplement in the diet, and in the traditional medicine, exhibiting numerous nutraceutical or pharmacological properties including anti-inflammatory, antioxidant, anti-cancer, hepatoprotective, neuroprotective, hypoglycaemic, and blood lipid-reducing functions.^(8)^ Most of the aforementioned health-promoting properties are related to the presence of glucosinolates (GSLs) and isothiocyanates (ITCs).^(9)^ The major GSL of the plant, namely 4-(*α*-L-rhamnosyloxy)benzyl GSL (glucomoringin, GMG) is the precursor of bioactive 4-(*α*-Lrhamnosyloxy)benzyl ITC (moringin, MG) released upon hydrolysis catalysed by the enzyme myrosinase (*β*-thioglucoside glucohydrolase; EC 3.2.1.147) at neutral pH (Figure 1). Many beneficial effects of MG have been especially ascribed to the activation of various detoxification enzymes and the reduction of certain inflammatory markers.^(10,11)^ Recently, the efficacy of *M. oleifera* seeds to mitigate myocardial damage was investigated in mice subjected to isoproterenol (ISP)-induced MI. Results showed improved survival rate and cardiac function of MI mice, proving anti-apoptosis and antioxidant properties of *M. oleifera* seed powder added to food.^(12)^ Noteworthy, *M. oleifera* seed is a remarkable source of bioactive MG as it contains GMG in high concentration up to 10%.^(10)^ Purified MG is a solid, odourless compound, which is stable at room temperature and can be conveniently prepared in large amount from GMG. This distinguishes MG from other natural bioactive dietary ITCs, such as renowned allyl ITC (2-propenyl ITC) from mustard, and *R*-sulforaphane ((*Rs*)-4-methylsulfinylbutyl ITC, *R*-SF) from Tuscan black kale and broccoli, which are volatile oils with pungent smells, when obtained in pure form. Despite their biological relevance, ITCs are generally characterised by poor water solubility and are often considered labile, gradually degrading in aqueous solutions, a limitation that complicates the assessment of their biological properties in both *in vitro* and *in vivo* studies. ^(13)^ However, these limitations can be effectively addressed through complexation of ITCs with cyclodextrins (CDs). In buffered/aqueous solution, the α-cyclodextrin/moringin (α-CD/MG) inclusion complex has recently proven enhanced water solubility and stability of MG by forming a stable inclusion system.^(10,14)^

The rat model of ISP-induced MI can be used as a well-standardised model because the pathophysiological changes following two consecutive ISP (85 mg/kg) doses administration occurring in rats are comparable to those taking place in human MI. Hence, it is widely used as an experimental model for the evaluation of cardio-protective effects of various natural and synthetic cardioprotective agents.^(15)^

Given this premises, the objective of the present work was to investigate the cardio- and neuroprotective effect of MG in a rat model of ISP induced MI.^(12,15)^ Aiming at providing a reference for its potential application in the prevention of MI damages, MG was administered in two different ways to overcome its lability: as a freshly prepared solution bioactivating purified GMG with myrosinase right before animals treatment, and as its improved stable formulation α-CD/MG inclusion complex.

### Materials and methods

### Chemicals

Acetonitrile and protease inhibitor (PI) cocktail were purchased from Nacalai Tesque (Kyoto, Japan). Formic acid was from Fisher Scientific (USA), ISP from Santa Cruz Biotecnology (USA), and phosphate buffer solution (PBS) from Elabscience (Texas, USA). All other chemicals were of the highest purity commercially available. All biochemical assays and enzyme-linked immunosorbent assays (ELISA) kits were purchased from Elabscience (Texas, USA).

### Glucomoringin and myrosinase purification

GMG was isolated from *M. oleifera* seeds (cake powder PKM-2 provided by Indena India Pvt. Ltd; Bangalore, India) and purified in two sequential steps, by anion exchange and size exclusion chromatography, according to a well-established procedure available at our labs and already reported extensively in a previous study.^(16)^ The purity was assessed by high-performance liquid chromatography (HPLC) analysis of the desulfo-derivative according to the EU standard procedure ISO 9167:2019 method,^(17)^ yielding about 99% based on peak area value and more than 95% on weight basis due to its marked hygroscopic properties.

Myrosinase was purified as already reported^(16)^ and stored at 4°C in sterile saline solution at neutral pH until use. The specific activity of the stock solution used in the present study was 60 U/mg of soluble protein and the enzymatic activity was 34 U/ml. One myrosinase unit was defined as the amount of enzyme able to hydrolyse 1 μmol/min of sinigrin at pH 6.5 and 37 °C.

### Preparation of moringin and α-cyclodextrin moringin complex for animal studies

*Fresh MG solution*. MG solution for *in vivo* animal study was freshly prepared through GMG bioactivation with myrosinase enzyme immediately before rat administration. Purified GMG powder was dissolved in PBS solution pH 7.2 and hydrolysed with myrosinase at 37 °C for 15 min (20 mg/kg GMG + 30 µl myrosinase/rat; 2.0 ml) yielding MG quantitatively, as previously reported.^(16)^

*MG powder.* MG powder for *α*-cyclodextrin inclusion was produced according to a reported method.^(18)^ Briefly, GMG was hydrolysed with myrosinase in 0.1 M phosphate buffer pH 6.5 at 37 °C. After monitoring the quantitative conversion of pure GMG into MG by HPLC, acetonitrile was added to the mixture up to a final concentration of 20%, and afterwards MG was purified by reverse-phase chromatography. Fractions containing MG were pooled together and freeze-dried yielding a white powder.

*α-CD/MG complex*. The α-CD/MG complex was prepared as previously reported.^(10)^ Briefly, 589 mg of MG powder were added to a clear solution of 2.21 g of α-CD in 65 mL of water. The mixture was then stirred at room temperature for 24 h, yielding a pale-yellow solution. The final solution was filtered with 0.45 µm filter and freeze-dried, affording 2.41 g of α-CD/MG complex with an equimolar ratio of the two constituents. The α-CD/MG complex was characterised by NMR and MS spectrometry.

### HPLC monitoring of glucomoringin conversion to moringin

In both experiment involving the production of MG as a fresh solution, as well as a powder for the making of α-CD/MG complex, the complete hydrolysis of GMG was confirmed by HPLC-PDA (Thermo Scientific Dionex, Ultimate 3000) equipped with a Acquity UPLC BEH C18 (100 mm × 2.1 mm, 1.7 µm) column. Chromatography was performed with 0.4 ml/min flow rate at 35 °C by eluting with a linear gradient of water (A) and acetonitrile (B) from 30% B to 80% in 20 min. Elution of MG was detected by a PDA, monitoring the absorbance at 229 nm^(16)^.

### Experimental protocol

*Animals.* We studied a total of 32 healthy male, 2.5-month-old Sprague-Dawley rats weighing 300-350 g at the start of the experiments (Universiti Putra Malaya, Malaysia). Rats were housed in plastic cages with wood shavings as bedding material at 22–24 °C in a well-ventilated animal house with a 12 h dark – 12 h light cycle. Chow pellets (24% protein, 65% carbohydrate, 11% fat, supplied by Altromin, Germany) and tap water were available *ad libitum* throughout the study. Rats were acclimatised for a period of 7 days. A simple randomisation was used to distribute rats into 4 groups, each cage containing two to three rats. Rats were treated via oral gavage once daily for 1 week.^(19,20)^

*MG dose*. Since the human intake of MG, to our knowledge, has not been reported, the dose of MG employed in the present study was based on a previous research examining the well-renowned ITC *R,S*-sulforaphane (*R,S*-SF). In that study authors investigated the role of *R,S*-SF (5 mg/kg, equivalent to 28.2 µmol/kg) in the attenuation of pathological cardiac remodelling after MI in rats.^(21)^

*Isoproterenol induced myocardial infarction*. MI was induced in male Sprague-Dawley rats by subcutaneous injection of ISP. In our experimental design, two injections of ISP (85 mg/kg) diluted in 2 ml of 0.9% saline were performed on days 6 and 7, 24 h apart. ISP was administered to all groups except the control one. False induction was achieved in the control group with administration of 2 ml of 0.9% saline on days 6 and 7.^(19,22)^

### Experimental groups

Animal grouping and number of animal per group was based on a similar previous study. Noteworthy, a positive control was not used in this study design. Using the minimum number of rats per group, it was possible to reduce the total number of animals.^(23)^

1. Control group (*N* = 8): rats not subjected to ISP;
2. MI group (*N* = 8): rats were pretreated daily for 7 days with 2 ml PBS pH 7.2. MI was induced on day 6 and day 7. ISP was administered at the dose of 85 mg/kg/d diluted in 2 ml of 0.9% saline with an interval of 24 h between applications;
3. MG + MI group (*N* = 8): rats were pretreated daily for 7 days with MG solution freshly prepared through GMG bioactivation with myrosinase (20 mg/kg GMG + 30 µl myrosinase/rat yielding 33.6 µmol/kg of MG; 2 ml PBS pH 7.2). On days 6 and 7, MI was induced by ISP administration in 2 ml of 0.9% saline at the dose of 85 mg/kg/d with an interval of 24 h between applications. Pretreatment with MG continued during day 6 and 7, with a 2-hour interval before the administration of ISP;
4. α-CD/MG + MI group (*N* = 8): rats were pretreated daily for 7 days with α-CD/MG (42 mg/kg, containing 32.7 µmol/kg of MG; 2 ml PBS pH 7.2). On days 6 and 7, MI was induced by ISP administration in 2 ml of 0.9% saline at the dose of 85 mg/kg/d with an interval of 24 h between applications. Pretreatment with MG continued during day 6 and 7, with a 2-hour interval before the administration of ISP.

All treatments were administered in the morning between 9–11am in the home cages. Rats were treated numerically from 1 to 8 and identified with tails marks.

After the treatment week, on day 8, animals were weighted, hemodynamic parameters were recorded, and behavioural tests were performed. All animals were then euthanised with ketamine/xylazine (60 mg/kg + 10 mg/kg i.p.) cocktail and then sacrificed. Blood, hearts and brains were sampled and processed for biochemical and histopathological studies.

### Collection of serum and preparation of tissue

Whole blood was collected in plain vacutainers, allowed to settle and then centrifuged at 5000 rpm for 15 min at 4°C to separate serum. The serum was aliquoted and stored at −80°C for quantitative determination of serum cardiac markers cTnI and CK-MB. Heart and brain from four rats of each group were dissected out on ice, rinsed in cold PBS and dried with absorbent paper. Fragments of left ventricle of the heart and the hippocampus were stored in cryotubes at −80°C and used for biochemical assays and ELISA after homogenisation. Samples of heart and brain were fixed in 10% buffered formalin solution for histopathologic analyses.^(22)^

### Preparation of heart and brain tissue homogenates

Fragments of left ventricle of the heart and the hippocampus were weighed and homogenised in ice cold PBS/EDTA/PI cocktail to prepare 10% tissue homogenates.^(22)^ After centrifugation at 3000 rpm for 10 min, the supernatant was used to assess oxidative stress markers including malondialdehyde (MDA), superoxide dismutase (SOD) and catalase (CAT). In addition, dopamine and serotonin were assayed in the hippocampal homogenate. Protein content of the homogenates was assayed using bicinchoninic acid (BCA) method.

### Hemodynamic parameter measurements

On day 8, 24 h after the last dose of ISP, heart rate (HR), systolic and diastolic blood pressure (SBP and DBP) and mean arterial pressure (MAP) were recorded using non-invasive blood pressure system (CODA, Kent Scientific) provided by Agro Biotechnology Institute (ABI, Malaysia). Rats were acclimatised on the CODA machine for 3 consecutive days prior to taking readings.^(24)^ The procedure involved insertion of the rat tail in the BP cuffs (O-cuff and VPR-cuff) while the upper body was restrained in a holder for stabilisation. Up to 10 accepted cycles/rat were used for analyses.

### Behavioural test

Non-invasive behavioural evaluations were made with the Open Field Test (OFT) to test behaviour and general motor function. A plywood floor (80 × 80 cm) divided by lines into 16 squares (20 × 20 cm) and elevated 40 cm above the ground was used.^(3)^ Rats were placed at the centre and allowed to acclimatise for 2 min. Videos of their behaviour were recorded for 3 min with the aid of a camera situated above the OFT box. The test was conducted individually starting 24 h after the second ISP injection, in the light cycle phase, dim light, between 9 am and 2 pm.^(3,25)^ To prevent rats from being distracted, the surface was treated with 70% ethanol solution after each animal test. Evaluations included number of line crossing, frequency of rearing (vertical stands), total distance travelled and total ambulatory time.^(3,6,25)^

### Biochemical analysis

*Serum cardiac markers.* The level of cTnI and the activity of CK-MB were measured in the serum by means of a highly specific enzyme immunoassay using commercially available ELISA kits and ELISA micro-plate reader (BioTek, Winosski, USA).^(22)^ Estimations were based on manufacturer protocols.

*Protein concentration.* 10% homogenates of myocardium and hippocampal samples were diluted and assayed for total protein using the BCA assay kit. The amount of protein was determined from the standard curve of bovine serum albumin by measuring the absorbance at 562 nm.^(3)^

*Malondialdehyde.* MDA level was estimated by the thiobarbituric acid reagent (TBAR) assay. Tissue homogenates were mixed with TBAR, incubated in boiling water for 15 min, cooled, and then centrifuged at 3000 rpm for 10 min. The supernatant was used to measure the absorbance at 532 nm. MDA level was expressed as nmol/g protein.^(3)^

*Catalase.* CAT activity was determined via colorimetric assay based on hydrogen peroxide (H_2_O_2_) and ammonium molybdate. Changes in absorbance were recorded spectrophotometrically measuring at 405 nm. CAT activity was expressed as units per mg of protein. One unit of CAT activity represents 1 µmol H_2_O_2_ decomposed per min at 37°C.^(22)^

*Superoxide dismutase.* SOD activity was determined via the water-soluble tetrazolium salt (WST-1) method based on formazan dye measuring the absorbance at 450nm.^(26)^ SOD activity was expressed as units per mg protein. One unit of SOD is defined as the amount of the enzyme required to produce 50% inhibition of formazan dye at 37°C.

*Dopamine.* Dopamine in the hippocampus was quantified using ELISA kit based on manufacturer protocol. Briefly, 10% brain homogenate was added to precoated ELISA plate together with biotinylated detection antibody and incubated for 45 min. Streptavidin-HRP, substrate reagent and stop solution where added sequentially and absorbance was read at 450 nm. Values were expressed as pg/mg protein.^(5)^

*Serotonine.* Serotonine in the hippocampus was quantified using ELISA kit based on manufacturer protocol. Briefly, 10% brain homogenate was added to precoated ELISA plate together with biotinylated detection antibody and incubated for 45 min. Streptavidin-HRP, substrate reagent and stop solution where added sequentially and absorbance was read at 450 nm. Values were expressed as pg/100 mg protein.^(5)^

### Histopathological examination

Heart and brain samples were fixed in 10% PBS-buffered formalin and 5 μm sections were prepared across the cardiac apex and hippocampus region, respectively. Sections were stained with haematoxylin and eosin (H&E) displaying the cytoplasm of cells in pink and the nucleus in blue. Microscopic observations were conducted with light microscope (Leica DM IL microscope, Germany), at 40x magnification for both heart and brain specimens. Results were rated semi-quantitatively into the following degrees based on intensity of alterations including cellular swelling, interstitial swelling, haemorrhage, oedema with inflammatory cell infiltrates, pyknosis:^(5)^ (0) no change, (1) mild - focal cellular damage or small multifocal degeneration with slight degree of inflammation, (2) moderate - extensive cellular degeneration and/or diffuse inflammatory process, (3) severe - necrosis with diffuse inflammatory process.^(20)^ The total histopathological score recorded for each group was compared among the different groups.^(5,27)^

### Statistical analyses

Data were analysed and presented as mean value with standard error of the mean (SEM) using the Minitab statistical software version 2019 (USA). Statistical analysis was performed using one-way analysis of variance to evaluate significant differences between the experimental groups, and the levels of significance were reported at *p* < 0.05 using Tukey’s post hoc test. To ensure a minimum of three biological replicates, four rats were selected using a simple random sampling following pretreatment and induction of MI.

## Results

### Bioactivation and quantification of MG

In order to activate the precursor GSL right before administration to the rats, GMG was hydrolysed with myrosinase and the quantitative formation of the bioactive ITC MG was confirmed by HPLC.^(16)^

### Production and characterisation of α-CD/MG

The α-CD/MG complex was prepared according to our previous description, and it was characterised by NMR and mass spectrometry. The protocol for the preparation of α-CD/MG was initially developed and described by Roselli *et al*.^(28)^ and further mentioned in several articles.^(14,29)^ Our NMR studies of α-CD/MG agreed with the recent paper reporting the characterisation of the inclusion complex.^(10)^ Moreover, the α-CD/MG complex infused in the electrospray source operating in negative ionisation mode (ESI-) provided the expected [α-CD/MG-H]- ions at m/z 1282.40.

### Animal analysed and adverse events

All rats were in good health prior to the experiments. To ensure a minimum of three biological replicates, four animals per group were selected for analysis, unless otherwise specified. Deceased animals were excluded. Pretreatment with MG and α-CD/MG did not induce any signs of toxicity. No mortality was observed in the control group, while the MI group exhibited a mortality rate of 2 out of 8 rats. In contrast, only 1 out of 8 rats died in both the MG + MI and α-CD/MG + MI pretreatment groups.

### Effect of MG and α-CD/MG pretreatment on heart weight, body weight and heart weight to body weight ratio

ISP administration did not cause a significant change in body weight (BW) (*p* = 0.425; Table 1) across groups. However, it led to a significant increase in heart weight (HW) (1.44 (SEM 0.08) *vs* 1.04 (SEM 0.06), *p* < 0.001) and the HW/BW ratio (HW/BW) (0.42 (SEM 0.02) *vs* 0.30 (SEM 0.02), *p* < 0.001) in MI rats compared to controls. Pretreatment with MG and α-CD/MG significantly reduced both HW and HW/BW compared to the MI group (*p* < 0.05 and *p* < 0.001, respectively).

**Table 1.** Changes in heart weight, body weight and heart weight to body weight ratio of male Sprague–Dawley rats used for the study. Control rats were injected daily only with saline (2 ml, sc). MI group rats were injected with isoproterenol (85 mg/kg, sc) on days 6 and 7 with a 24 h interval. MG + MI group was pretreated with 20 mg/kg GMG + 30 µl myrosinase/rat for 7 days. α-CD/MG + MI group was pretreated with 42mg/kg α-CD/MG for 7 days. Both pretreated groups were injected with isoproterenol (85 mg/kg, sc) on days 6 and 7 with a 24 h interval. The parameters were measured on day 8, 24 h after the second dose of isoproterenol. Data are presented as mean values and standard error of the mean (SEM) of 6 measurements performed on 6 rat samples for each studied group

	Control		MI		MG + MI		α-CD/MG + MI	
Parameters	Mean	SEM	Mean	SEM	Mean	SEM	Mean	SEM
HW (g)	1.04	0.06	1.44**	0.08	1.30#	0.03	1.19##	0.02
BW (g)	343.00	6.42	345.94^$^	6.67	352.18^$^	5.89	344.44^$^	4.02
HW/BW	0.30	0.02	0.42**	0.02	0.37##	0.02	0.34##	0.02

HW, heart weight; BW, body weight; HW/BW, heart weight to body weight ratio.

Mean values were significantly different compared to the control group (Tukey’s post hoc test): **P < 0.001.

Mean values were significantly different compared to the MI group (Tukey’s post hoc test): #*P* < 0.05, ##*P* < 0.001.

Mean values were not significantly different compared to the control group (Tukey’s post hoc test): ^$^no significant difference between groups.

### Cardioprotective effect of MG and α-CD/MG on hemodynamic parameters

MI rats demonstrated a significant increase in HR and significant decreases of SBP, DBP, and MAP (*p* < 0.001). Pretreatment with MG and α-CD/MG significantly reduced HR (*p* < 0.05 and *p* < 0.001, respectively), while also improving SBP, DBP, and MAP (Table 2).

**Table 2.** Cardioprotective effect of pretreatment with MG and α-CD/MG on hemodynamic parameters heart rate, systolic blood pressure, diastolic blood pressure, mean arterial pressure of male Sprague–Dawley rats used for the study. Control rats were injected daily only with saline (2 ml, sc). MI group rats were injected with isoproterenol (85 mg/kg, sc) on days 6 and 7 with a 24 h interval. MG + MI group was pretreated with 20 mg/kg GMG + 30 µl myrosinase/rat for 7 days. α-CD/MG + MI group was pretreated with 42mg/kg α-CD/MG for 7 days. Both pretreated groups were injected with isoproterenol (85 mg/kg, sc) on days 6 and 7 with a 24 h interval. The parameters were measured on day 8, 24 h after the second dose of isoproterenol. Data are presented as mean values and standard error of the mean (SEM) of 6 measurements performed on 6 rat samples for each studied group

	Control		MI		MG + MI		α-CD/MG + MI	
Parameters	Mean	SEM	Mean	SEM	Mean	SEM	Mean	SEM
HR (BPM)	338.71	4.0	465.96**	4.34	382.08#	3.34	345.00##	15.7
SBP (mmHg)	129.68	1.53	77.82**	1.67	102.42#	1.97	115.30##	3.16
DBP (mmHg)	82.08	0.70	60.25**	2.10	73.01#	2.09	75.03#	1.96
MAP (mmHg)	97.95	0.39	66.11**	1.94	82.81#	0.90	88.45#	1.60

HR, heart rate; BPM, beats per min; SBP, systolic blood pressure; DBP, diastolic blood pressure; MAP, mean arterial pressure.

Mean values were significantly different compared to the control group (Tukey’s post hoc test): **P < 0.001.

Mean values were significantly different compared to the MI group (Tukey’s post hoc test): #*P* < 0.05, ##*P* < 0.001.

### Neuroprotective effect of MG and α-CD/MG on behavioural parameters

**Figure 2.**
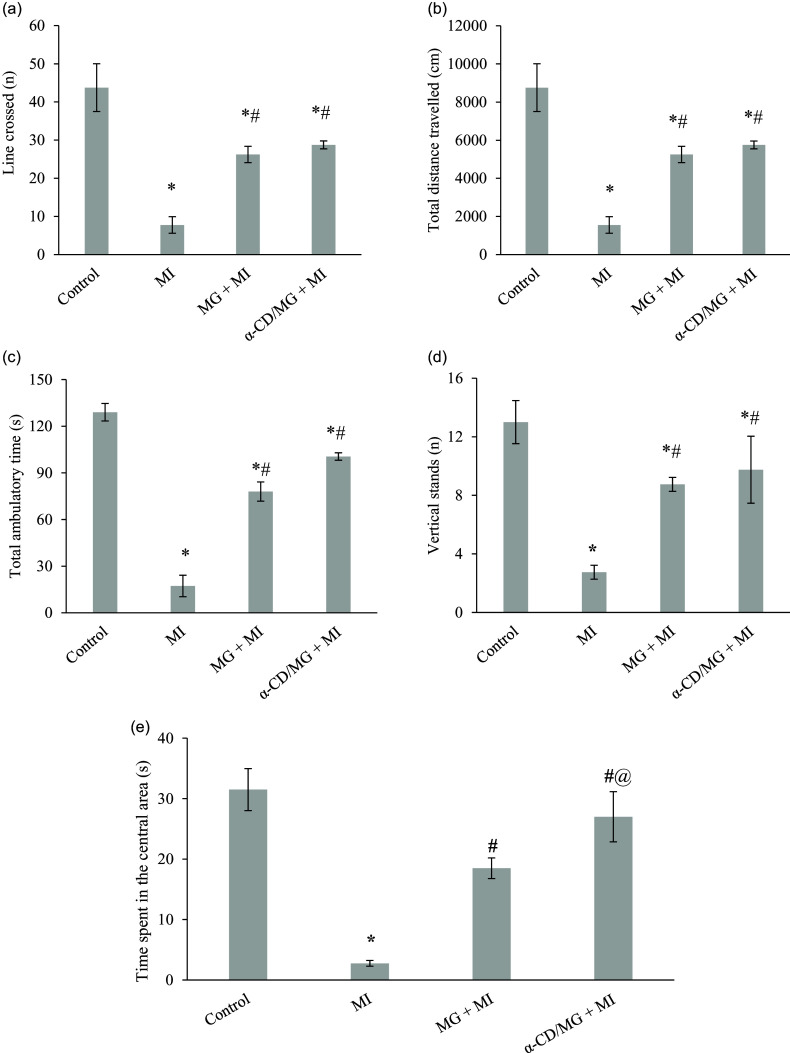
Neuroprotective effect of moringin (MG) and α-cyclodextrin/moringin complex (α-CD/MG) on behavioural parameters of rat groups. (a) Line crossed, (b) total distance travelled, (c) total ambulatory time, (d) vertical stands, (e) time spent in central area. Data are presented as mean values and standard error of the mean of 6 measurements performed on 6 rat samples for each studied group. MG + MI and α-CD/MG + MI represent groups pretreated with MG and α-CD/MG complex, respectively. Mean values were significantly different compared to the control group (Tukey’s post hoc test): *P < 0.05. Mean values were significantly different compared to the MI group (Tukey’s post hoc test): #*P* < 0.05. Mean values were significantly different compared to the MG + MI group (Tukey’s post hoc test): @*P* < 0.05.

OFT analyses revealed significant behavioural deficits in the MI group, including reduced line crossings, total distance travelled, ambulatory time, number of vertical stands, and time spent in the central area, compared to controls (Figure 2). Pretreatment with MG and α-CD/MG significantly improved all these parameters relative to the MI group. Notably, α-CD/MG pretreatment resulted in a significantly increased time spent in the central area compared to MG pretreatment.

### Cardioprotective effect of MG and α-CD/MG on serum cardiac markers

**Figure 3.**
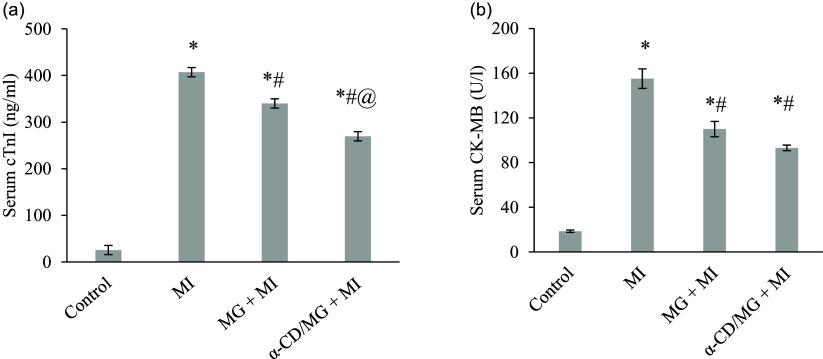
Cardioprotective effect of moringin (MG) and α-cyclodextrin/moringin complex (α-CD/MG) on serum cardiac injury markers of rats. (a) Serum cardiac troponin I (cTnI), (b) and serum creatine kinase myocardial band (CK-MB). Data are presented as mean values and standard error of the mean of 4 measurements performed on 4 rat samples for each studied group (justified by the need for a minimum of 3 biological replicates and animal welfare considerations). MG + MI and α-CD/MG + MI represent groups pretreated with MG and α-CD/MG complex, respectively. Mean values were significantly different compared to the control group (Tukey’s post hoc test): *P < 0.05. Mean values were significantly different compared to the MI group (Tukey’s post hoc test): #*P* < 0.05. Mean values were significantly different compared to the MG + MI group (Tukey’s post hoc test): @*P* < 0.05.

MI rats exhibited significantly elevated serum levels of cTnI and CK-MB (*p* < 0.05). In contrast, pretreatment with MG and α-CD/MG significantly reduced both biomarkers (*p* < 0.05 *vs* MI group; Figure 3). Notably, α-CD/MG pretreatment was more effective than MG alone in reducing cTnI levels.

### Cardioprotective effect of MG α-CD/MG on markers of oxidative stress

**Figure 4.**
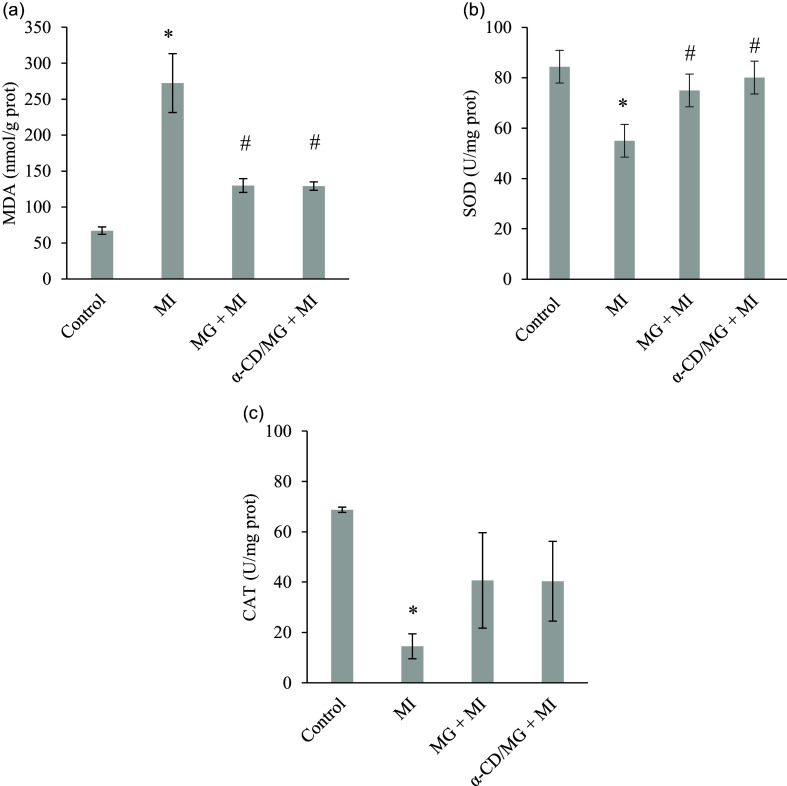
Cardioprotective effect of moringin (MG) and α-cyclodextrin/moringin complex (α-CD/MG) on myocardial oxidative stress markers of rats with isoproterenol induced MI: (a) malondialdehyde (MDA), (b) superoxide dismutase (SOD), (c) catalase (CAT). Data are presented as mean values and standard error of the mean of four measurements performed on 4 rat samples for each studied group. MG + MI and α-CD/MG + MI represent MG and α-CD/MG pretreated groups, respectively. Mean values were significantly different compared to the control group (Tukey’s post hoc test): *P < 0.05. Mean values were significantly different compared to the MI group (Tukey’s post hoc test): #*P* < 0.05.

MI significantly increased myocardial MDA levels (*p* < 0.05) and decreased the activity of antioxidant enzymes SOD and CAT (*p* < 0.05). Pretreatment with MG and α-CD/MG significantly lowered MDA and restored SOD levels (*p* < 0.05), while CAT levels showed a non-significant increase (*p* > 0.05; Figure 4).

### Cardioprotective effect of MG α-CD/MG on myocardial morphology

**Figure 5.**
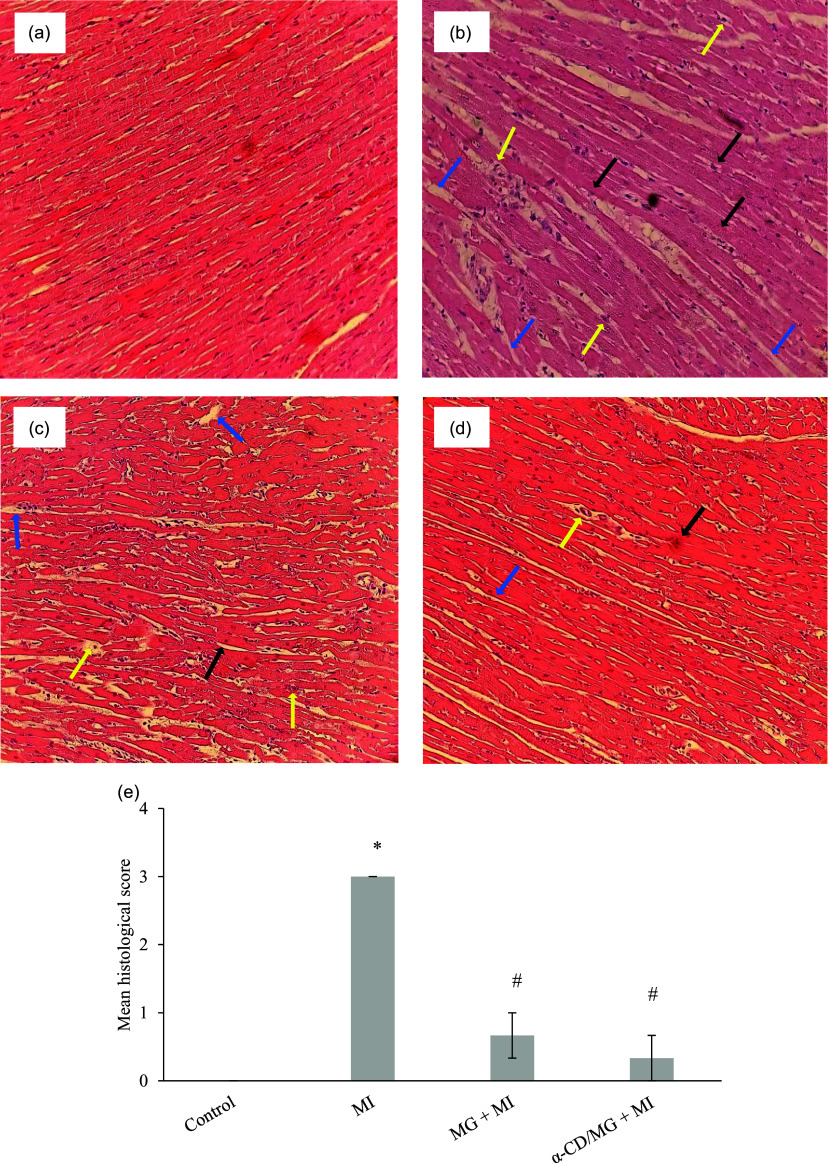
Representative haematoxylin and eosin (H&E) stained section of hearts of the different study groups photographed at 40× magnification. (a) Control group showed normal myocardial architecture with intact cell membrane without necrosis, (b) MI group showed diffused necrosis with inflammatory patches, (c) MG + MI group showed patchy areas of mild necrosis, neutrophilic infiltration and moderate increase in intercellular spaces, (d) α-CD/MG + MI group showed near normal cardiac musculature with visible cross striations and slight increase in the intercellular spaces. Blue arrows indicate intermuscular oedema, black arrows indicate myocyte necrosis, and yellow arrows indicate neutrophil infiltration. (e) Mean histological scores were calculated based on the degree of cellular infiltration, oedema, haemorrhage, and necrosis. Data are presented as mean value and standard error of the mean (SEM) of four measurements performed on four rat samples per group. MG + MI and α-CD/MG + MI represent MG and α-CD/MG pretreated groups, respectively. *P < 0.05 *vs* control group; #*P* < 0.05 *vs* MI group (Tukey’s post hoc test).

H&E-stained sections (Figure 5a,b,c,d) from the MI group revealed pronounced myocardial damage, including necrosis, inflammatory infiltration, oedema, and haemorrhage. These pathological features were markedly attenuated in the MG + MI and α-CD/MG + MI groups. The α-CD/MG + MI group displayed myocardial architecture comparable to controls, with only mild oedema and few necrotic cells. Histological scores were significantly lower in both pretreated groups compared to MI rats (*p* < 0.05; Figure 5e).

### Hippocampal oxidative stress markers

**Figure 6.**
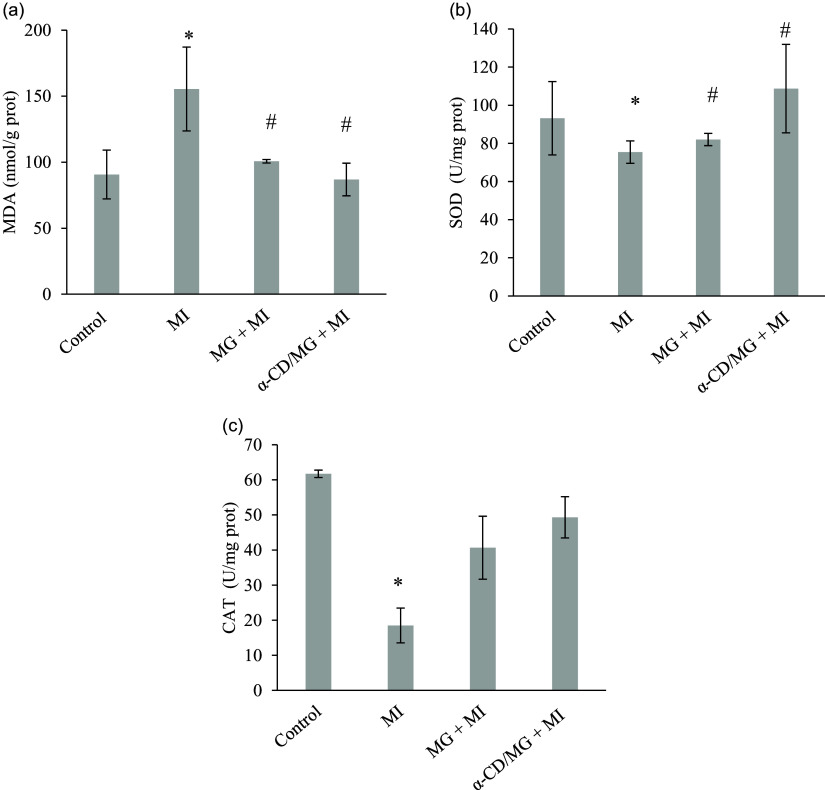
Neuroprotective effect of moringin (MG) and α-cyclodextrin/moringin complex (α-CD/MG) on hippocampal oxidative stress markers of rats with isoproterenol induced MI: (a) malondialdehyde (MDA), (b) superoxide dismutase (SOD), (c) catalase (CAT). Data are presented as mean values and standard error of the mean of 4 measurements performed on 4 rat samples for each studied group. MG + MI and α-CD/MG + MI represent MG and α-CD/MG pretreated groups, respectively. Mean values were significantly different compared to the control group (Tukey’s post hoc test): *P < 0.05. Mean values were significantly different compared to the MI group (Tukey’s post hoc test): #*P* < 0.05.

MI induced significant oxidative stress in the hippocampus, evidenced by elevated MDA and reduced SOD and CAT activities (*p* < 0.05). Pretreatment with MG and α-CD/MG significantly reduced MDA levels and improved SOD activity. CAT activity increased, although not significantly (Figure 6).

### Hippocampal monoamines

**Figure 7.**
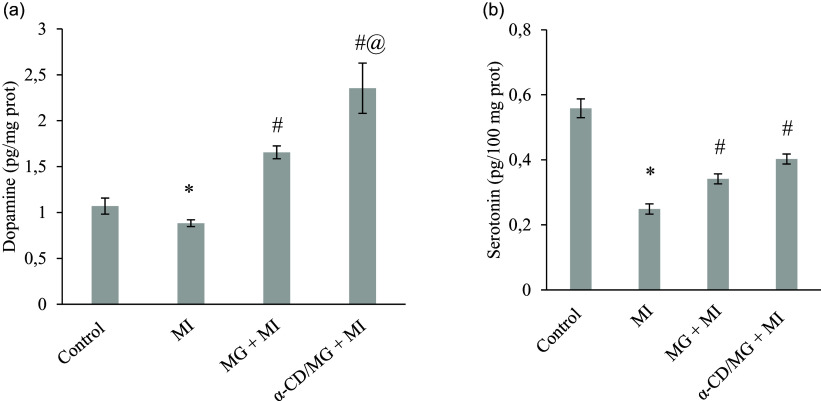
Neuroprotective effect of moringin (MG) and α-cyclodextrin/moringin complex (α-CD/MG) on hippocampal monoamines of rats with isoproterenol induced MI: (a) dopamine, (b) serotonin. Data are presented as mean values and standard error of the mean of 4 measurements performed on four rat samples for each studied group. MG + MI and α-CD/MG + MI represent MG and α-CD/MG pretreated groups, respectively. Mean values were significantly different compared to the control group (Tukey’s post hoc test): *P < 0.05. Mean values were significantly different compared to the MI group (Tukey’s post hoc test): #*P* < 0.05. Mean values were significantly different compared to the MG + MI group (Tukey’s post hoc test): @*P* < 0.05.

ISP significantly decreased hippocampal dopamine and serotonin levels in MI rats (*p* < 0.05). Pretreatment with MG and α-CD/MG significantly restored these neurotransmitters (p < 0.05). α-CD/MG pretreatment produced significantly higher dopamine levels than MG alone and even higher than control values (Figure 7).

### Hippocampal morphology

**Figure 8.**
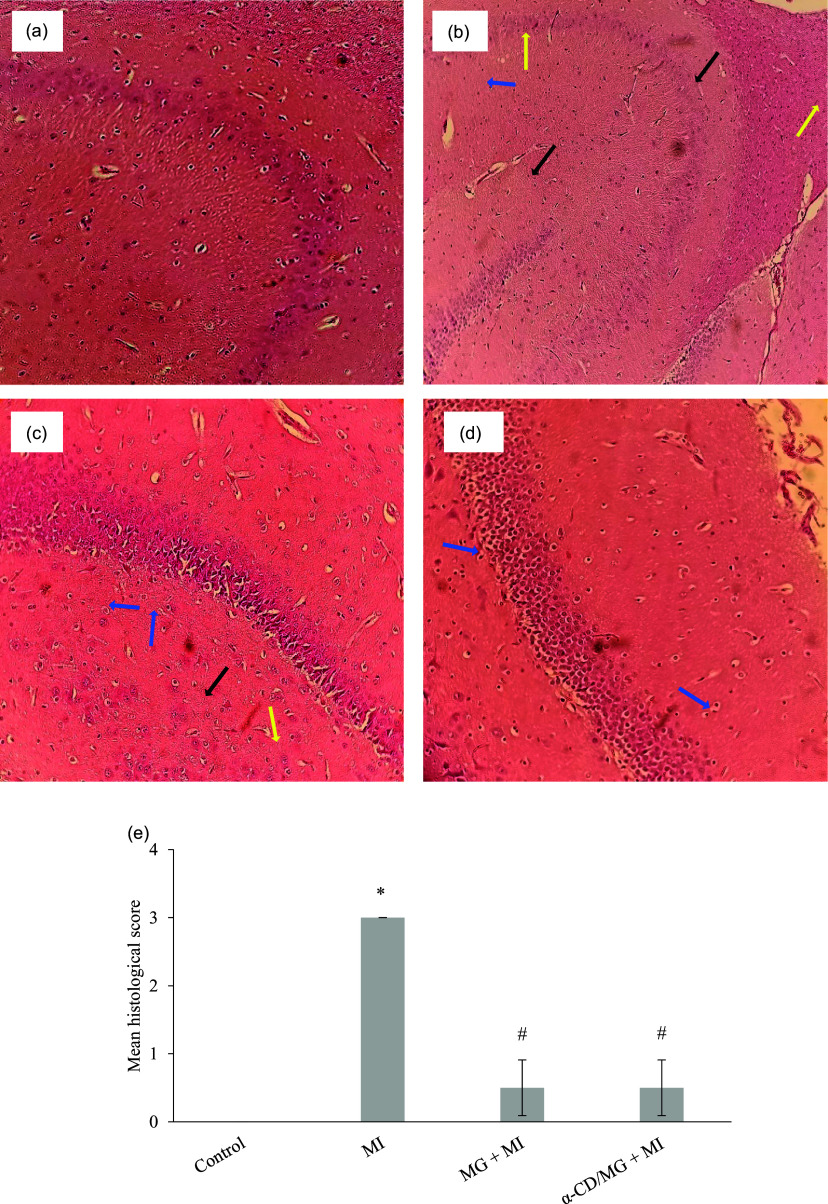
Representative haematoxylin and eosin (H&E) stained section of hippocampus of the different study groups photographed at 40× magnification. (a) Control group showed normal morphology of the hippocampus, (b) MI group showed oedema, pyknotic neurons, inflammatory cells, engorged blood vessels, (c) MG + MI group showed few inflammatory cells and moderate oedema, and (d) α-CD/MG + MI group showed few neutrophils and mild oedema. Blue arrows indicate neuronal oedema, black arrows indicate pyknotic neurons, and yellow arrows indicate neutrophil infiltration. (e) Mean histological score were calculated based on the extent of cellular infiltration, oedema and neuronal swelling. Data are presented as mean value and standard error of the mean (SEM) of four measurements performed on four rat samples per group. MG + MI and α-CD/MG + MI represent MG and α-CD/MG pretreated groups, respectively. *P < 0.05 *vs* control group; #*P* < 0.05 *vs* MI group (Tukey’s post hoc test).

The MI group showed significant histopathological alterations in the hippocampus, including neutrophil infiltration, vascular congestion, oedema, and nuclear pyknosis. These abnormalities were significantly reduced in both MG + MI and α-CD/MG + MI groups (*p* < 0.05), with α-CD/MG offering greater protection (Figure 8).

## Discussion

The therapeutic use of dietary ITCs is grounded in their ability to counter oxidative stress, inflammation, and apoptosis - key pathogenic mechanisms underlying both cardiovascular and neurodegenerative diseases.^(7,30)^ Accumulating evidence suggests that the protective effects of *Moringa oleifera* are largely attributable to its ITC content, particularly MG, which is readily isolated from the seeds of the plant.^(1)^ MG has previously demonstrated efficacy in various models of neurological disorders, including Alzheimer’s disease, multiple sclerosis, Parkinson’s disease, and malignant astrocytoma.^(29)^ MI manifests with both cardiovascular and neurobehavioral symptoms, which are often addressed separately in clinical practice. Given the documented antioxidant activity of MG and its inclusion complex with α-CD, this study evaluated their potential protective effects in a rat model of ISP-induced MI. ISP is a synthetic catecholamine known to produce infarct-like myocardial necrosis in experimental animals and it is commonly used to assess cardiac dysfunction.^(20,22)^

Rats in the MI group exhibited significantly increased HW and HW/BW ratios compared to controls, consistent with myocardial oedema, mucopolysaccharide accumulation, and cellular infiltration beginning within hours of MI induction.^(24,29)^ Pretreatment with MG and α-CD/MG significantly reduced both HW and HW/BW, with α-CD/MG demonstrating a more pronounced effect.^(20,25)^ This reduction may reflect decreased inflammatory cell recruitment and reduced oedema, as previously shown in models of LPS-induced sepsis and carrageenan-induced inflammatory oedema.^(31)^

To evaluate the cardioprotective effects of MG and α-CD/MG, hemodynamic parameters were measured using a non-invasive CODA system. ISP administration resulted in elevated heart rate and reduced blood pressures, attributable to oxidative damage and myocardial oedema.^(30)^ Even minimal increases in myocardial water content can significantly impair cardiac function.^(20)^ Pretreatment with MG and α-CD/MG ameliorated these hemodynamic changes, likely through preservation of myocardial membrane integrity and endogenous antioxidant defenses.^(14,31)^ The effect was more substantial in the α-CD/MG group.

Behavioural assessments using the OFT revealed that MI rats displayed anxiety-like behaviours, characterised by reduced locomotor and exploratory activities. These effects are consistent with reduced cerebral perfusion and altered catecholamine release.^(25,32)^ The MI group also had reduced MAP values, supporting the link to impaired tissue perfusion. Pretreatment with MG and α-CD/MG improved behavioural parameters significantly. Previous studies have shown similar improvements in locomotor function with MG pretreatment in models of multiple sclerosis.^(16)^ Furthermore, cerebral ischaemia following cardiac events has been associated with cognitive impairments involving hippocampal function.^(33)^ The improved MAP and behavioural outcomes observed here suggest enhanced cerebral perfusion as a mechanism of neuroprotection.^(25,34)^

Cardiac biomarkers such as cTnI and CK-MB, which are typically absent from circulation under normal conditions, were significantly elevated following ISP administration, indicating myocardial damage.^(20,35)^ Pretreatment with MG and α-CD/MG significantly reduced these markers, suggesting maintenance of membrane integrity and inhibition of mitochondrial apoptotic pathways.^(36,37)^ While other markers such as CK and LDH have shown responsiveness to cardioprotective agents, cTnI remains the most sensitive indicator of MI.^(35,38)^

Oxidative stress plays a central role in MI pathophysiology.^(39)^ ISP administration increases lipid peroxidation and reduces myocardial antioxidant capacity.^(20,24)^ In this study, MI was associated with elevated MDA and reduced SOD and CAT. Pretreatment with MG and α-CD/MG reversed these changes, particularly in the α-CD/MG group (Figure 4). Similar antioxidant responses have been observed with melatonin and rhapontigenin.^(20,40)^ MG and α-CD/MG likely exert these effects by reducing ROS production, inhibiting lipid peroxidation, and enhancing endogenous antioxidant systems via the Nrf2 pathway.^(14,41,42)^

Histopathological analysis revealed classical signs of MI in ISP-treated rats, including myofibrillar necrosis, inflammation, and nuclear degeneration. These alterations are attributed to oxidative stress and inflammation.^(43)^ MG and α-CD/MG pretreatment reduced these pathological features, indicating protective effects on myocardial structure. These findings are in line with studies involving sulforaphane,^(44)^ and indole-3-cabinol,^(43)^ where histological damage was mitigated.

Parallel findings were observed in the hippocampus. Due to its metabolic demands and susceptibility to oxidative stress, the hippocampus showed elevated MDA and depleted SOD and CAT in MI rats.^(6,25,45)^ Pretreatment with MG and α-CD/MG attenuated these effects. This aligns with *in vitro* studies on differentiated SH-SY5Y cells, where MG demonstrated free radical scavenging activity,^(46)^ and with *in vivo* models treated with ghrelin and ginkgolide B.^(6,25)^

MI also impaired hippocampal dopamine and serotonin levels, consistent with oxidative disruption of neurotransmitter synthesis pathways.^(4)^ MG and α-CD/MG pretreatment restored these monoamines, suggesting mitigation of redox imbalance and improved neurotransmitter synthesis. MG is known to enhance dopaminergic signalling and preserve neuronal integrity.^(37)^ Improvements in exploratory behaviour likely reflect these neurochemical changes, as dopamine plays a crucial role in motivation and motor function.^(47)^ The restoration of hippocampal monoamines may thus contribute to the behavioural improvements and offer protection against MI-associated neuropsychological impairment.^(33)^ Histological examination of the hippocampus further supported the neuroprotective effects of MG and α-CD/MG. MI caused oedema, vascular congestion, and neuronal pyknosis, which were ameliorated by pretreatment. These results are consistent with studies in neuroinflammatory models of amyotrophic lateral sclerosis and multiple sclerosis, where MG reduced histological damage.^(16,42)^ Our findings also align with previous reports showing decreased neuronal density and increased astrocytosis in MI-affected hippocampi, reversible with antioxidant treatment.^(25)^

It is important to note that it is unlikely that MG or α-CD/MG exert their effects through direct inhibition of ISP. Complete prevention of MI features would be expected if antagonism occurred. However, while MG and α-CD/MG pretreatment attenuated damage, residual pathology persisted. For example, although cardiac biomarkers and histological damage were reduced, they were not eliminated. This partial protection indicates a modulatory, rather than inhibitory, mechanism.

Furthermore, mortality was only partially reduced (1/8 *vs* 2/8), indicating that ISP retained its pharmacological activity. The consistent oxidative stress pattern - elevated MDA, reduced SOD/CAT - and its normalisation by the pretreatment support the hypothesis that MG and α-CD/MG enhance endogenous defence mechanisms rather than antagonise ISP directly.

Mechanistically, the cardioprotective and neuroprotective effects of MG and α-CD/MG appear to be mediated primarily through their strong antioxidant and anti-inflammatory properties. MG is known to activate the Nrf2 signalling pathway, which upregulates the expression of endogenous antioxidant enzymes such as SOD and CAT, thereby enhancing the cell ability to neutralise ROS and to reduce lipid peroxidation.^(14,41,42)^ Additionally, MG has been reported to inhibit proinflammatory pathways, including NF-κB, thereby reducing cytokine-driven inflammatory responses that contribute to myocardial and neuronal injury.^(10,14,29)^ The α-CD/MG formulation further improves MG stability and bioavailability, thereby enhancing its ability to reach target tissues and exert protective effects. These molecular mechanisms support the observed reduction in MDA levels, restoration of monoamine levels, and preservation of histological integrity in both myocardium and hippocampus. Collectively, these findings suggest that MG and α-CD/MG confer protection not by directly antagonising isoproterenol (ISP), but by enhancing tissue resilience through modulation of oxidative stress and inflammation-associated signalling pathways.

Taken together, this study demonstrates the cardioprotective and neuroprotective effects of MG and α-CD/MG in a rat model of ISP-induced MI. α-CD/MG exhibited superior efficacy compared to MG alone. Hemodynamic improvements were more pronounced with α-CD/MG, correlating with reduced oedema and preserved myocardial integrity. Behavioural tests showed significant reversal of anxiety-like symptoms, associated with enhanced cerebral perfusion. Cardiac biomarkers and oxidative stress parameters were normalised, and histological integrity in both myocardium and hippocampus was preserved. These results suggest that the enhanced bioactivity of MG as an inclusion complex with α-CD^(10)^ contributes to its therapeutic potential.

Overall, MG and α-CD/MG represent promising bioactive agents for the prevention or attenuation of MI-related cardiac and neurological damage.

## Conclusions

This is the first study to evaluate the protective effects of the bioactive compound moringin (MG), the principal dietary isothiocyanate derived from *Moringa oleifera*, in a rat model of isoproterenol-induced myocardial infarction, an *in vivo* model that replicates key pathological features of human MI. Our findings demonstrate that both freshly prepared MG and its α-cyclodextrin inclusion complex (α-CD/MG) confer significant cardioprotective and neuroprotective effects. Pretreatment with these agents effectively reduced myocardial and hippocampal oxidative stress, lowered serum cardiac injury markers, preserved hippocampal monoamine levels, and improved behavioural outcomes indicative of reduced anxiety.

These beneficial effects are likely mediated by the antioxidant properties of MG and α-CD/MG. However, the precise molecular pathways through which these agents exert their protective actions remain to be elucidated. Furthermore, comprehensive toxicological assessments are essential before considering their potential application in clinical settings for myocardial infarction prevention or management.

Collectively, the results presented here support the prophylactic potential of MG and α-CD/MG as promising bioactive compounds for mitigating both cardiac and neurological damage associated with myocardial infarction.
